# Effects of job rotation and role stress among nurses on job satisfaction and organizational commitment

**DOI:** 10.1186/1472-6963-9-8

**Published:** 2009-01-12

**Authors:** Wen-Hsien Ho, Ching Sheng Chang, Ying-Ling Shih, Rong-Da Liang

**Affiliations:** 1 Dept. of Medical Information Management, College of Health Science, Kaohsiung Medical University, 100, Shih-Chuan 1st Road, Kaohsiung, 80708, Taiwan; 2 Dept. of Nursing & Nutrition Support Team, Kaohsiung Medical University Hospital, 100, Shih-Chuan 1st Road, Kaohsiung, 80708, Taiwan; 3 Dept. of Marketing and Logistics Management, National Penghu University, Taiwan

## Abstract

**Background:**

The motivation for this study was to investigate how role stress among nurses could affect their job satisfaction and organizational commitment, and whether the job rotation system might encourage nurses to understand, relate to and share the vision of the organization, consequently increasing their job satisfaction and stimulating them to willingly remain in their jobs and commit themselves to the organization. Despite the fact that there have been plenty of studies on job satisfaction, none was specifically addressed to integrate the relational model of job rotation, role stress, job satisfaction, and organizational commitment among nurses.

**Methods:**

With top managerial hospital administration's consent, questionnaires were only distributed to those nurses who had had job rotation experience. 650 copies of the questionnaire in two large and influential hospitals in southern Taiwan were distributed, among which 532 valid copies were retrieved with a response rate of 81.8%. Finally, the SPSS 11.0 and LISREL 8.54 (Linear Structural Relationship Model) statistical software packages were used for data analysis and processing.

**Results:**

According to the nurses' views, the findings are as follows: (1) job rotation among nurses could have an effect on their job satisfaction; (2) job rotation could have an effect on organizational commitment; (3) job satisfaction could have a positive effect on organizational commitment; (4) role stress among nurses could have a negative effect on their job satisfaction; and (5) role stress could have a negative effect on their organizational commitment.

**Conclusion:**

As a practical and excellent strategy for manpower utilization, a hospital could promote the benefits of job rotation to both individuals and the hospital while implementing job rotation periodically and fairly. And when a medical organization attempts to enhance nurses' commitment to the organization, the findings suggest that reduction of role ambiguity in role stress has the best effect on enhancing nurses' organizational commitment. The ultimate goal is to increase nurses' job satisfaction and encourage them to stay in their career. This would avoid the vicious circle of high turnover, which is wasteful of the organization's valuable human resources.

## Background

Job rotation inspires nurses to achieve higher performance, allowing continuous growth at work, extended knowledge and skill, and increasing clinic patient care-taking quality. Scholars have all proposed that job rotation may help employees to acquire multiple capabilities and expand vision, and that it can be an approach to reduce job burnout [1-3]. But emotional pressure often occurs in the work environment where interpersonal interactions are highly involved [4-6]. Especially, the nurses working at hospitals not only implement independent and professional nursing activities in accordance with doctors' advice, but take responsibility for any immediate threat to patients' lives as well. Thus, the importance of nurses is undeniable, and the influence of nurses' qualities and capabilities on medical care quality can never be ignored [7,8]. Therefore, the primary concern of the practical field of medical care is to exhaustively recognize how role stress among nurses could affect their job satisfaction and organizational commitment, and effectively utilize the job rotation system to enhance and develop nurses' job satisfaction and organizational commitment, in order to promote competitive advantages.

### Literature Review and Research Hypotheses

#### Job rotation

Job rotation is also called cross-training, meaning an employee of a unit or department can learn diversified job skills during a specific period of time; it is also regarded as a practical approach to enrich and expand job assignments [9]. Therefore, job rotation is also planned on-the-job training for cultivating future candidates of management by transferring a management trainee from one department to another to increase his or her understanding and credentials in all aspects [3]. In addition, job rotation is also regarded as a method of job design that, on top of allowing employees to learn job skills from different departments, eliminates employee fatigue caused by tedious job assignments by changing such assignments; the challenge of these new assignments can encourage an employee's enthusiasm once again, and improve employee morale to increase output [10,11]. When implementing job rotation, the quality of an individual's work experience should be focused on, instead of quantity. Organizations should arrange the next rotation plan according to each employee's learning capability and adjustment time [12]. Therefore, high frequency of job rotation may not be better; factors such as employee's background, learning status, and job familiarity should be taken into consideration for frequency of job rotation.

The definition of job rotation here refers to a professional job cross training plan that helps employees expand their job territory while broadening their working experience and skills, stimulating their working spirit and cultivating their interpersonal relationships by shifting medical personnel to different departments or units of the same department. In reality, job rotation means neither job promotion nor paid adjustment. The structure of this study was based on one rotation as the only variable. The task was mainly to obtain the opinions and inclinations of nursing personnel toward job rotation. The purpose is to discuss hospital nursing personnel's comments and opinions on the implementation of job rotation.

#### Role stress

[4,5] suggested that a role is the manifestation of behavior appropriate to an individual's position. In an organization, an individual's role stress refers to "the stress formed by the combined expectations of an individual's behavior from all circles." While facing role stress, an individual may produce unfavorable behavior to an organization, such as performance reduction, job burnout, and resignation, which deserve to be taken seriously. In the role theory, [13] divided role stress into two types: role conflict and role ambiguity. [14] further separated "role overload" from role conflict, so that there are three types of role stress, which are role ambiguity, role conflict, and role overload [15]. [16], on the other hand, asserted that role stress is composed of five constructs, including role ambiguity, role overload, role conflict, role incongruity, and role incompetence or role over-qualification. This study adopts the viewpoint of role ambiguity, role conflict, and role overload as the three types of role stress.

#### Job satisfaction

[17] believed job satisfaction is a positive or negative attitude that an employee has toward his or her job or some specific aspects of the job, and is an internal state of mind of an individual. [18] pointed out it is a feeling or affection held by a member of an occupation system; if the feeling is positive or the response is active, then the member is satisfied, and vice versa. [19] proposed that job satisfaction is an employee's feeling about his or her work environment, which includes the job itself, supervisor, work group, organization, and life. [20,21] suggested that the level of job satisfaction depends on the difference between what a person actually gains from his or her job and what he or she expects. [22] proposed that job satisfaction is the level in which an employee likes or dislikes his or her job. [23] also pointed out that job satisfaction is an employee's feeling about his or her job and is a general attitude derived from an evaluation of all aspects in a job.

#### Organizational commitment

[24,25] indicated that organizational commitment is an individual's willingness to dedicate efforts and loyalty to an organization. The commitment to a job is because an individual believes the cost of leaving an organization is so high, that it is difficult for the individual to leave after consideration of the investment and sacrifice made to the organization [26,27]. Psychological commitment proposed by [28] is also called normative commitment. [29] explained that this type of organizational commitment means the members of an organization have active and highly positive inclination toward the organization, and such inclination includes identification with an organization's goals and values, dedication to a job, and loyalty to an organization. [30,31] described the process of the forming of organizational commitment with investment and dedication that an individual shall develop a relatively deep identification with an organization after such individual has invested in the organization at a relatively high level, and a forced commitment to the organization is consequently formed. [32,33] pointed out that basically organizational commitment is a structural phenomenon of a transaction between an individual and an organization, and its non-transferable investment result shall increase as time goes on; members are reluctant to leave the organization because of the salary, status, position, and friendship among colleagues.

### Relationships among job rotation, job satisfaction, and organizational commitment

In regard to the relationship between job satisfaction and organizational commitment, [34-36] pointed out in their study of job satisfaction and organizational commitment that the two have significant positive correlation [37,38]. [10,39,19] proposed that job rotation has the advantage of increasing job satisfaction. [12,40] also believed implementing job rotation has the advantage of increasing organizational commitment in the aspect of employees' career and emotion. From the above statements, the following hypotheses are formed:

***Hypothesis 1: Nurses' job rotation has a positive influence on job satisfaction***.

***Hypothesis 2: Nurses' job rotation has a positive influence on organizational commitment***.

***Hypothesis 3: Nurses' job satisfaction has a positive influence on organizational commitment***.

### Relationships among role stress, job satisfaction, and organizational commitment

The results of the research of [24,41] showed that there is a significant negative correlation between role stress and organizational commitment, and role conflict, role ambiguity, and role overload have the most remarkable influence in the construct of role stress [42,5]. In regard to the relationship between role stress and job satisfaction, [15,5] found that the tension at work caused by role ambiguity, role conflict, and role overload has a significant negative correlation with job satisfaction. From the above statement, the following hypotheses are formed:

***Hypothesis 4: Nurses' role stress has a negative influence on job satisfaction***.

***Hypothesis 5: Nurses' role stress has a negative influence on organizational commitment***.

## Methods

### Research Framework (Model)

**Figure 1:**
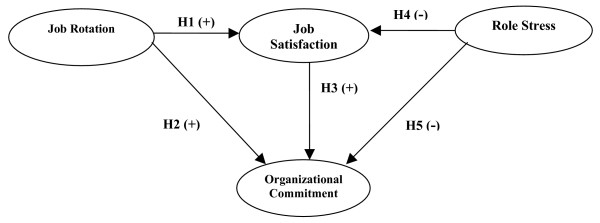
**Conceptual framework of the relationship among job rotation, job satisfaction, organizational commitment, and role stress**.

Our conclusion from the motivation, purpose, and literature review is that nurses' job rotation has a positive influence on job satisfaction and organizational commitment, nurses' job satisfaction has a positive influence on organizational commitment, and role stress among nurses has a negative influence on their job satisfaction and organizational commitment. Therefore, in this study, job rotation and role stress among nurses are independent variables, and organizational commitment is a dependent variable, while job satisfaction is the mediating variable, in order to discuss the correlation among all the variables. The overall research framework is shown as Figure 1.

### Research Subject and Sampling Method

There are two reasons for the job rotation of nurses: 1. The planned rotation: for example, in compliance with the promotion of nurses in basic units or training of associate head nurses; 2. Non-planned rotation: for instance, large-scale temporary transfer of personnel to different sections and offices in a unit (due to support for other units, dismissal, leave of absence and so on). Generally, the period for the first type of rotation is a half year and for the second type is about three months.

In order to survey a diverse and representative sample of RNs, nursing staff in two large and influential hospitals (each was a teaching hospital and medical center, and each had more than 1200 sickbeds) in southern Taiwan were surveyed. We first visited the management supervisors at the hospitals in person to acquire approval. Volunteer supervisors of nurses were asked to post on bulletin boards at their respective units a notice for recruiting volunteer nurses who would participate in the anonymous questionnaire. The response period was limited to two months. A seminar was delivered to the supervisors of the nurses to explain the details, and an introductory letter was attached to the questionnaire to explain the motivation of this study and guarantee the respondents' confidentiality. Anyone who was also interested in learning about the result of this study was able to request a copy through the contact address provided in the questionnaire. 650 copies of the questionnaire were only distributed (Medical Center A: 340 copies; Medical Center B: 310 copies) to those nurses who had had job rotation experience, and 550 were collected between October 2006 and December 2006, among which 18 were rejected due to incomplete answers. 532 valid copies were collected (Medical Center A: 273 copies; Medical Center B: 259 copies), and the valid response rate was 81.8%.

### Study Tools

The following is the explanation of the questionnaire which comprised questions already developed in foreign studies and modified to serve the study purposes. First, dimensions of questionnaire forms were obtained from the literature and used to compile questionnaires. Second, the dimensions were slightly modified to create initial questionnaires based on the research purposes and industry features. Third, tests were repeatedly administered to three academic professors in the industry, and to two medical specialists and five nurses with experience of job rotation; a pilot run of the questionnaire was administered to 38 nurses. Thirty-two valid questionnaire forms meeting the acceptable standard of more than thirty recommended by [43] were collected, and the analytical results indicated that all factor loadings were greater than 0.7, and all Cronbach's α values exceeded 0.7, therefore, none of the items were deleted [43]. Finally, the questionnaire was officially released. Deserving of special attention is that, the questionnaire employed did not allow the measurement of job rotation levels, and thus, cannot establish whether participants experienced job rotation or not.

The questionnaire employed a 5-point Likert scale from 1, for "strongly disagree" to 5, for "strongly agree." Table 1 summarizes constructs and variables, including operational definitions for all variables. Questionnaires were examined for reliability and validity as follows:

**Table 1:** Summary of Constructs and Variables

**Construct**	**Variable**	**Operational Definition**	**References**
**Job Rotation**	Job Rotation	Hospital nursing personnel transfer among departments of different functions or different units of the same department without promotion or salary adjustment.	Campion et al. (1994) & Anil and Brian (2004)

**Role Stress**	Role Ambiguity	Insufficient information about work objectives to be completed and the uncertainty of others' expectations toward an individual and the results generated after objectives have been accomplished.	Kahn et al. (1964), Piko (2006), & Van Sell, Brief, and Schuler (1981)
	Role Conflict	Under the conditions of an individual's time, resources, capability, or value, it (role conflict) may take place if the conditions are inconsistent with the standards, criteria, and expectations set by role senders.	
	Role Overload	Psychological burden that is formed when a role sender has overly high requirement and expectations for a role recipient's job, which exceeds what the role recipient is capable of, from a legitimated position.	

**Job Satisfaction**	Internal Satisfaction	The opportunities to demonstrate abilities, sense of achievement obtained from work, ethical values of the work, opportunities to provide services.	Judge and Bono (2001) & Best and Thurston (2004)
	External Satisfaction	Job content, salary, unobstructed channels for promotion, work environment and equipment.	

**Organizational Commitment**	Value Commitment	Strong beliefs in and acceptance of the organizational objectives and values.	Porter et al. (1974) & Trimble (2006)
	Effort Commitment	Willingness to dedicate more efforts for the organizational benefits.	
	Retention Commitment	Willingness to stay in the organization as a member of the organization.	

1. Reliability analysis: Principal component factor analysis was used to extract major contributing factors, and varimax of the orthogonal rotation was performed to maximize the differences in factor loading carried by every common factor after the rotation to help recognize common factors. Thus, as Table 2 illustrates, the analytical results indicated that factor loadings were 0.79 to 0.92 meeting the acceptable standard of more than 0.7, and all Cronbach's α values exceeded 0.7 [43,44].

**Table 2:** Results of Reliability Analyses

**Construct**	**Factor Naming**	**Cronbach's α**
**Job rotation**		
	Job rotation	**0.92**
**Role Stress**		
	Role Ambiguity	**0.79**
	Role Conflict	**0.82**
	Role Overload	**0.81**
**Job Satisfaction**		
	Internal Satisfaction	**0.88**
	External Satisfaction	**0.89**
**Organizational Commitment**		
	Value Commitment	**0.82**
	Effort Commitment	**0.88**
	Retention Commitment	**0.85**

2. Construct convergent validity (confirmatory factor analysis): The confirmatory factor analysis could gain higher recognition than expert content validity [43], and the results for all dimensions were listed in Table 3. All of the adequacy indicators met the acceptable standard recommended by [43]. Parameter (λ) between each latent variable and manifest variable were estimated to determine the significance of the estimated parameter (λ) in order to evaluate convergent validity. Thus, as Table 4 shows, the t values for the factor loading of all measurement items reached the level of significance (p < 0.01), no single factor included only one question, and the composite reliability values for all constructs were greater than 0.6, which demonstrated satisfactory convergent validity [45,43].

**Table 3:** Results of Convergent Validity Analysis

**Indicator**	**Job Rotation**	**Role Stress**	**Job Satisfaction**	**Organizational Commitment**
χ2/df. (< 3)	2.96	2.47	2.55	2.30
GFI (> .9)	0.90	0.93	0.91	0.93
AGFI (> .8)	0.82	0.87	0.83	0.85
NFI (> .9)	0.91	0.90	0.91	0.93
RMSR (< .08)	0.03	0.02	0.03	0.02

*Note*. χ^2^/df. = Ratio of Chi-square, GFI = Goodness of Fit Index, AGFI = Adjusted GFI, NFI = Normal Fit Index, RMSR = Root Mean Square of Standardized Residual.

**Table 4:** Confirmatory Factor Analysis of all the Constructs

**Construct**	**Variable/Question Item**	**Standardized Loading**	**Measurement Error**	**Composite Reliability**	**AVE**
**Job Rotation**	1. I believe job rotation is a type of job training. **(JRT)**	0.40**	0.84	**0.75**	**0.73**
	2. Job rotation broadens my knowledge and skill in other fields. **(JRK)**	0.66**	0.56		
	3. I am willing to accept job rotation now. **(JRA)**	0.52**	0.73		
	4. Before job rotation, the organization seeks my consent. **(JRC)**	0.78**	0.39		
	5. I believe job rotation is an excellent system. **(JRS)**	0.64**	0.59		
	6. Overall, I like job rotation. **(JRL)**	0.76**	0.42		

**Role Stress**	**Role Ambiguity (RA)**			**0.78**	**0.75**
	1. I do now know how to utilize my time appropriately.	0.77**	0.41		
	2. I have no idea of what I have to do every day.	0.80**	0.36		
	3. I have no clue of what the hospital's expectations of my job are.	0.83**	0.31		
	**Role Conflict (RC)**			**0.72**	**0.71**
	4. Others often have inconsistent requirements for my job.	0.69**	0.52		
	5. I often did some unnecessary work.	0.72**	0.48		
	6. Sometimes the tasks the hospital assigned to me were too difficult or too complicated.	0.76**	0.42		
	**Role Overload (RO)**			**0.81**	**0.76**
	7. My everyday workload is too much for me to finish.	0.82**	0.33		
	8. My assignments seem to become more and more complicated.	0.81**	0.34		
	9. I am in charge of many duties and tasks at the same time, which are too much for me to handle.	0.78**	0.39		

**Job Satisfaction**	**Internal Satisfaction (IS)**			**0.86**	**0.77**
	1. My job provides me the chance to fulfill my ability.	0.79**	0.37		
	2. I can derive a sense of achievement from my job.	0.82**	0.32		
	3. I am satisfied with my self-development from my job.	0.86**	0.27		
	4. I am competent to do my present job.	0.91**	0.18		
	5. I find my job meaningful.	0.79**	0.37		
	
	**External Satisfaction (ES)**			**0.85**	**0.79**
	6. I am satisfied with my present job's environment and facilities.	0.79**	0.38		
	7. Compared with other nursing personnel, I am content with my salary.	0.84**	0.29		
	8. My present job provides me a chance for promotion.	0.87**	0.25		
	9. I get along well with my colleagues.	0.74**	0.45		
	10. I would get complimented when I do well on my job.	0.78**	0.39		

**Organizational Commitment**	**Value Commitment (VC)**			**0.79**	**0.79**
	1. I have a strong sense of belonging to the hospital.	0.58**	0.53		
	2. I am willing to serve this hospital.	0.71**	0.49		
	3. I am proud to be a part of this hospital.	0.81**	0.34		
	4. I care about the future development of the hospital.	0.70**	0.51		
	
	**Effort Commitment (EC)**			**0.80**	**0.77**
	5. I am willing to put extra effort to achieve the goals of my job.	0.82**	0.33		
	6. I try my best to overcome the difficulties of my job.	0.84**	0.29		
	7. I am willing to pass on my working experience to new staff.	0.55**	0.56		
	8. I actively help my colleagues to solve problems in their work.	0.71**	0.49		
	
	**Retention Commitment (RC)**			**0.78**	**0.75**
	9. I feel I will have a promising future if I stay in this hospital.	0.58**	0.54		
	10. I have a profound attachment to this hospital.	0.64**	0.59		
	11. With the present working environment and system, I am willing to stay in this hospital.	0.48**	0.77		
	12. If I leave this hospital, I will have guilt feelings.	0.81**	0.35		

### Data Analysis Method

Finally, the SPSS 11.0 and LISREL 8.54 (Linear Structural Relationship Model) statistical software packages were used for data analysis and processing, as follows:

1. Descriptive statistical analysis: To see the characteristics of samples.

2. Structural Equation Modeling (SEM): According to [46], structural equation modeling allows not only the determination of relationship extent between variables, but also the examination of chain of cause and effect. This means that results do not merely show empirical relationships between variables when defining the practical situation. For this reason, this study chose structural equation modeling to test hypotheses. This study also used several indices, including Chi-square ratio (< 3), goodness of fit index (GFI > .9), adjusted goodness of fit index (AGFI > .8), normal fit index (NFI > .9) and root mean square of standardized residual (RMSR < .08) to evaluate overall model fitness.

### Ethical Considerations

The study had been verbally approved by each institution's ethics committee before the research started and before the supervisors were approached. Nursing staff supervisors were asked to post a notice requesting volunteers for the anonymous questionnaire on bulletin boards at their respective units. The nurse supervisor made the questionnaires available for volunteer nurses to fill out anonymously on site; respondent nurses could ask questions directly to the supervisor, and the supervisors collected even those questionnaires not filled out on site. The response period was limited to two months. The authors invited all nursing supervisors to a seminar to explain the details of the study, and an introduction letter was attached to the questionnaire to explain the purpose of the study and to ensure respondent confidentiality. The questionnaire provided contact information so that respondents could later inquire about the results of the study.

## Results

### Characteristics of Samples

The demographic data revealed that 48.1% of participants were under 30, and 57.7% were unmarried. Subjects with less than junior college education comprised 44.2% of the study population. The employment data revealed that 30.3% had between three and six years of work experience. 93.8% were not currently in management positions and 50.8% were currently working in hospital wards (see Table 5).

**Table 5:** Descriptive Statistics of Sample (N = 532)

**Variable/Item**	**Frequency**	**Percentage**
**Hospitals**		
Medical Center A	**273**	**51.3**
Medical Center B	**259**	**48.7**
**Gender**		
Female	**532**	**100**
**Age**		
30 or under	**256**	**48.1**
31–40	**210**	**39.5**
41–50	**66**	**12.4**
**Marital status**		
Married	**225**	**42.3**
Not married	**307**	**57.7**
**Education**		
College or under	**235**	**44.2**
Bachelor	**226**	**42.5**
Master or above	**71**	**13.3**
**Seniority**		
Less than 3 years	**92**	**17.3**
3–6 years	**161**	**30.3**
6–10 years	**149**	**28.0**
10 years or above	**130**	**24.4**
**Units**		
Ward	**270**	**50.8**
Intensive care unit	**142**	**26.7**
Others	**120**	**22.5**
**Job title**		
Nurse	**499**	**93.8**
Head nurse	**33**	**6.2**

*Note*. ** p < .01, AVE = Average variance extracted.

### The Relationships among Nurses' Job Rotation, Role Stress, Job Satisfaction, and Organizational Commitment

**Figure 2:**
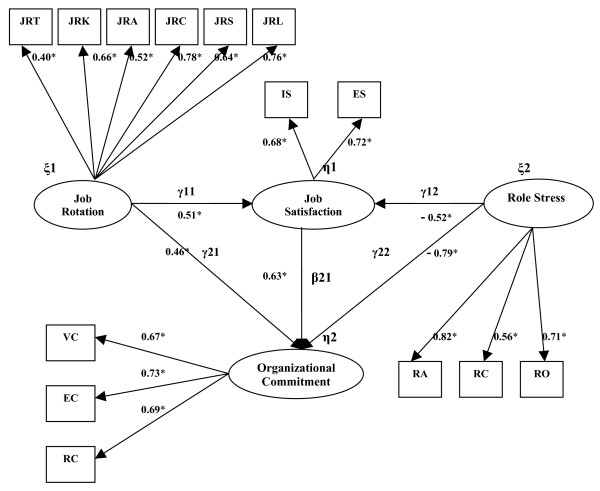
**Structural Equation Modeling (SEM) of the relationship among job rotation, job satisfaction, organizational commitment, and role stress**. (*Note*. * represents p < .01)

The Linear Structural Relationship Model was employed to examine the relationships among nurses' job rotation, role stress, job satisfaction, and organizational commitment [45,46]. Hypotheses 1 to 5 in this study were demonstrated to be significant, as in Figure 2. Nurses' job rotation had a positive influence on job satisfaction (γ_11 _= 0.51) and organizational commitment (γ_21 _= 0.46). Nurses' job satisfaction (β_21 _= 0.63) had a positive influence on organizational commitment. Nurses' role stress had a negative influence on job satisfaction (γ_12 _= -0.52) and organizational commitment (γ_22 _= -0.79). Figure 2 shows the SEM of this study and Table 6 shows the model fit goodness of the structural equation modeling. In short, it can be concluded that the research model is applicable for the data.

**Table 6:** Model Fit Goodness of the Structural Equation Modeling

**Fitness Statistics**	**χ ^2^/df**.	**GFI**	**AGFI**	**NFI**	**RMSR**
Standard Value	< 3	> .9	> .8	> .9	< .08
**Conceptual Model**	**2.15**	**0.93**	**0.89**	**0.91**	**0.026**

*Note*. χ^2^/df. = Ratio of Chi-square, GFI = Goodness of Fit Index, AGFI = Adjusted GFI, NFI = Normal Fit Index, RMSR = Root Mean Square of Standardized Residual.

## Discussion

### Conclusions and implications

According to the nurses' views, there are five major results in this study: (1) job rotation among nurses could have an effect on their job satisfaction; (2) job rotation could have an effect on organizational commitment; (3) job satisfaction could have a positive effect on organizational commitment; (4) role stress among nurses could have a negative effect on their job satisfaction; and (5) role stress could have a negative effect on their organizational commitment. The implications are discussed, as follows:

### Academic implications

Cases in which past researchers' viewpoints corresponded to the results of this study that job satisfaction has positive effects on nurses' organizational commitment [34,37]. Our findings also support the statement that job rotation could have an effect on nurses' job satisfaction and organizational commitment. This agrees with the assertions of previous relevant studies [12,40].

The results support that role stress exercises obviously negative influences on organizational commitment, this inference is in accordance with the assertions of certain scholars in the past [47,48,5]. The findings of this study also reveal that role stress exercises obviously negative influences on job satisfaction indicating that role stress among nurses negatively influences their job satisfaction. It is in accordance with the assertions of relevant studies in the past [49-51].

### Practical implications

Due to the fact that hospitals depend on nurses' work to operate and it takes tremendous time and effort to train nurses' talent, retaining excellent nurses and stimulating them to do their best to serve hospitals and take on future challenges are crucial issues for hospitals to stay competitive in today's environment. As a practical and excellent strategy for manpower utilization, a hospital could promote the benefits of job rotation to both individuals and the hospital while implementing job rotation periodically and fairly. The ultimate goal is to increase nurses' job satisfaction and encourage them to stay at their work. This would avoid the vicious circle of high turnover which is wasteful of the organization's valuable human resources.

The findings of this study reveal that role stress among nurses exercises negative influences on both job satisfaction and organizational commitment. The parameter estimates of the correlation show that the negative influence that role stress among nurses has on organizational commitment (-0.79) is greater than that on job satisfaction (-0.52). Therefore, when a medical organization attempts to enhance nurses' commitment to the organization, the findings suggest that reduction of role ambiguity (with a path coefficient of 0.82) in role stress has the best effect on enhancing nurses' organizational commitment.

Avian Influenza (AI) has caused hundreds of deaths in Europe. The hospital system of Taiwan, standing in the first line of prevention and treatment, has to effectively employ the experience of SARS in order to tackle the new serious challenge of AI. Though Taiwan is not an epidemic area of AI, it is important for us to be well prepared to confront it, given that prevention is more important than treatment. It is essential that we try our best to build up an efficiently preventive system for AI and other serious infectious diseases. This study aims to investigate what is most important and critical in the preventive system. More specifically, what are the priorities in such a preventive system, especially under a limited condition of budgets and human resources?

### Research Limitations

The findings of this study should be considered in view of the following limitations.

1. This study merely investigated the correlation between perception of job rotation, role stress, job satisfaction, and organizational commitment in a population of nurses. Factors influencing organizational commitment such as the nature of employment, work environment, work experience and management style, await further study.

2. This study examined a population of nurses in a single country and should be generalized cautiously to other populations. However, given the context of the study, the survey results exhibited adequate validity and reliability.

3. The primary research instrument in the study was the questionnaire, which has a certain degree of validity and reliability. However, the results of the voluntary survey would have been subject to numerous factors which could cause variations in the results, such as defensiveness, pretending, personal emotion and other attitudes.

4. This study examined only one time period, which would not reveal factors which have long-term effects. A multiple time period approach is suggested for follow-up study. Analyzing multiple periods of data would achieve more complete and objective statistical data.

## Competing interests

The authors declare that they have no competing interests.

## Authors' contributions

The following indicated the contributions of each author in this study: WHH conceived of the study, and participated in its design and coordination; CSC participated in the design of the study and performed the statistical analysis & FG, conceived of the study, and participated in its design and coordination; YLS conceived of the study, and participated in its design and coordination; and RDL conceived of the study, and participated in its design and coordination. All authors read and approved the final manuscript.

## Pre-publication history

The pre-publication history for this paper can be accessed here:


